# Efficacy and Safety of Intravitreal Faricimab in Age-Related Macular Degeneration—A Review

**DOI:** 10.3390/jcm14196712

**Published:** 2025-09-23

**Authors:** Chih-Cheng Chan, Pei-Kang Liu, Kai-Chun Cheng, Hung-Chi Lai, Yo-Chen Chang

**Affiliations:** 1 Department of Clinical Education and Training, Kaohsiung Medical University Hospital, Kaohsiung 807, Taiwan; jamesjan0614@gmail.com; 2 Department of Ophthalmology, School of Medicine, Kaohsiung Medical University, Kaohsiung 807, Taiwan; 990288@gap.kmu.edu.tw (P.-K.L.); pington64@gmail.com (K.-C.C.); 3 Department of Ophthalmology, Kaohsiung Medical University Hospital, Kaohsiung 807, Taiwan; phillip7678@gmail.com; 4 Department of Ophthalmology, Kaohsiung Municipal Siaogang Hospital, Kaohsiung Medical University, Kaohsiung 812, Taiwan

**Keywords:** faricimab, neovascular age-related macular degeneration, anti-VEGF, angiopoietin-2, efficacy, safety, durability, intraocular inflammation, treatment burden

## Abstract

Neovascular age-related macular degeneration (nAMD) is a significant cause of vision loss globally, with intravitreal anti-vascular endothelial growth factor (anti-VEGF) agents forming the cornerstone of treatment. Despite advances, the considerable treatment burden associated with frequent injections and the occurrence of suboptimal responses in some patients highlight an ongoing need for more effective and durable therapeutic options. Faricimab, a bispecific antibody that targets both VEGF-A and angiopoietin-2 (Ang-2), has been developed to address these challenges by promoting greater vascular stability and potentially offering extended treatment intervals. This review synthesizes current evidence from pivotal clinical trials (TENAYA/LUCERNE), real-world studies, meta-analyses, and case reports on the efficacy, durability, and safety of intravitreal faricimab for nAMD. Key efficacy outcomes, such as changes in best-corrected visual acuity and anatomical parameters (e.g., central subfield thickness, retinal fluid dynamics, pigment epithelial detachment morphology), are evaluated in both treatment-naïve and previously treated/treatment-resistant nAMD populations. The safety profile, including intraocular inflammation, retinal vasculitis, retinal pigment epithelium tears, and systemic adverse events, is also comprehensively addressed. Faricimab has demonstrated non-inferior visual outcomes compared to aflibercept 2 mg, alongside robust anatomical improvements and a significant potential for reduced treatment frequency, thereby lessening patient and healthcare system burden. While generally well-tolerated, ongoing monitoring for adverse events remains essential.

## 1. Introduction

Age-related macular degeneration (AMD) is a progressive, multifactorial neurodegenerative disease affecting the macular region of the retina, which is responsible for central, high-resolution vision [1,2]. It is a leading cause of irreversible vision loss and legal blindness in individuals over 50 years of age in developed countries, including Japan, and its prevalence is increasing globally [1,2,3,4]. The global burden of AMD is projected to affect nearly 300 million people by 2040 [2]. Specifically, in China, late-stage AMD prevalence ranges from 0.38% in those aged 45–49 to 3.88% in those 85–89 years old, potentially affecting over 55 million people by 2050 [5].

Neovascular AMD (nAMD), also known as wet AMD, constitutes about 10–15% of all AMD cases but is responsible for the majority (approximately 90%) of severe vision loss associated with the condition [1,2,6]. nAMD is characterized by the pathological growth of new blood vessels, termed macular neovascularization (MNV), which can originate from the choroid (choroidal neovascularization) or, less commonly, the retinal circulation [1,2]. These new vessels are typically fragile and prone to leakage, leading to the accumulation of intraretinal fluid (IRF) and subretinal fluid (SRF), intraretinal or subretinal hemorrhage, and the formation of pigment epithelial detachments (PEDs) [1,2,7]. If untreated, these exudative processes can cause irreversible damage to photoreceptors, submacular fibrosis, and profound, permanent loss of central vision [1,2]. The impact of nAMD on quality of life is substantial, affecting activities such as reading, driving, and facial recognition [8].

The identification of vascular endothelial growth factor A (VEGF-A) as a key pathogenic factor driving angiogenesis and vascular permeability in nAMD was a landmark discovery [1,2,9,10]. Consequently, intravitreal injections of anti-VEGF agents, such as ranibizumab, aflibercept, and off-label bevacizumab, became the standard of care, revolutionizing nAMD management by significantly improving visual outcomes for many patients [2,3,6,10,11,12,13]. These therapies, including aflibercept which demonstrated efficacy in the VIEW 1 and VIEW 2 studies, became the standard of care [3,6,8,12]. A Cochrane review confirmed the effectiveness of aflibercept compared to ranibizumab [13].

However, VEGF signaling is only one component of this complex pathophysiology [14]. Some patients exhibit persistent disease activity (e.g., residual fluid despite monthly injections) or require frequent injections (e.g., every 4–8 weeks) to maintain visual and anatomical gains [2,7,14,15,16]. For example, the Comparison of Age-Related Macular Degeneration Treatments Trials (CATT) showed that even with monthly ranibizumab, over 50% of patients had persistent macular fluid on optical coherence tomography (OCT) after two years [17]. This high treatment burden, encompassing numerous clinic visits and injections, can lead to undertreatment in real-world settings, diminished patient adherence, and significant socioeconomic strain on patients, their families, and healthcare systems [14,16,18,19,20]. Real-world data indicate that a substantial fraction of patients, potentially 19–27%, may require ongoing four-weekly anti-VEGF injections to control disease activity [15]. This highlights the unmet need for therapies that can provide more durable disease control and reduce treatment frequency [2,14].

The angiopoietin/tyrosine kinase with immunoglobulin and epidermal growth factor receptor-2 (Ang/Tie2) signaling pathway has gained recognition as another critical system involved in regulating vascular homeostasis and pathological angiogenesis [2,14,21]. Angiopoietin-2 (Ang-2), in particular, is upregulated in various retinal vascular diseases, including nAMD, and its levels have been shown to correlate with disease severity [2,14,22,23]. Ang-2 acts as a competitive antagonist of Angiopoietin-1 (Ang-1) at the Tie2 endothelial receptor, disrupting Ang-1’s stabilizing effects on blood vessels and promoting vascular instability [2,14,21,24]. This destabilization can lead to pericyte loss, increased vascular permeability, neovascularization, inflammation, and fibrosis, often synergistically with VEGF-A, but also through VEGF-independent mechanisms [2,14].

Faricimab (Vabysmo^®^, Roche/Genentech, San Francisco, CA, USA) is a novel bispecific antibody specifically designed for intraocular use, engineered to simultaneously and independently bind and neutralize both VEGF-A and Ang-2 [2,14,21,22,24,25]. This dual-targeting strategy aims to provide more comprehensive disease control by addressing two key pathogenic pathways, with the goal of enhancing vascular stability, reducing inflammation, and offering more durable treatment effects compared to anti-VEGF monotherapies, thereby potentially reducing treatment frequency [2,14,21,24]. This review will consolidate and discuss the current clinical evidence regarding the efficacy, durability, and safety of intravitreal faricimab in the management of nAMD.

For this narrative review, we searched PubMed/MEDLINE, Embase, and Scopus for articles published between January 2010 and September 2025 using combinations of the terms “age-related macular degeneration”, “faricimab”, “aflibercept”, “ranibizumab”, “anti-VEGF”, “artificial intelligence”, and “real-world”. Both pivotal clinical trials and real-world studies were included, and additional references were identified through bibliographic review. No formal inclusion or exclusion criteria were applied beyond relevance to the efficacy, durability, or safety of faricimab in nAMD.

## 2. Mechanism of Action and Pharmacology of Faricimab

Faricimab is a humanized bispecific antibody, based on an IgG1 isotype, with a molecular weight of approximately 149–150 kDa [2,21,24]. It is produced using CrossMab technology, which enables a single antibody molecule to possess two distinct antigen-binding fragments (Fabs) with different specificities [24]. One Fab arm of faricimab targets and neutralizes all isoforms of VEGF-A, while the other Fab arm is engineered to bind and neutralize Ang-2 [2,21]. The simultaneous binding to these two key mediators of nAMD pathology is intended to provide a more robust and synergistic effect in promoting vascular stability and reducing pathological neovascularization and leakage [14,26].

A significant molecular engineering aspect of faricimab is its modified Fc (fragment crystallizable) region [2,14,21,24,27]. Specific amino acid substitutions have been introduced in the Fc domain to abolish binding to neonatal Fc receptors (FcRn) and Fc gamma receptors (Fc gamma Rs) [2,14,22,24,27,28]. The elimination of FcRn binding is intended to reduce the systemic half-life of the antibody, thereby minimizing systemic exposure and potential systemic side effects [2,24,27]. The lack of Fc gamma R binding aims to prevent Fc-mediated effector functions, such as antibody-dependent cell-mediated cytotoxicity (ADCC) and complement-dependent cytotoxicity (CDC), which could theoretically reduce the risk of intraocular inflammation [2,24,27]. Recent studies have confirmed that faricimab does not bind to FcRn [29].

The therapeutic rationale for dual Ang-2 and VEGF-A inhibition is rooted in their distinct yet interconnected roles in the pathophysiology of retinal vascular diseases like nAMD [14]. VEGF-A is a primary driver of increased vascular permeability and angiogenesis [1,9,10]. Ang-2, often upregulated in pathological conditions including nAMD, acts as a context-dependent ligand for the Tie2 receptor on endothelial cells [2,14]. It competes with Ang-1, a Tie2 agonist that promotes vascular quiescence, maturation, and stability [2,14]. By antagonizing Ang-1/Tie2 signaling, elevated Ang-2 levels lead to vascular destabilization, pericyte detachment, endothelial barrier breakdown, and increased responsiveness of endothelial cells to VEGF-A and other pro-inflammatory and pro-angiogenic stimuli [2,14,21]. Ang-2 also has direct pro-inflammatory effects and is implicated in promoting fibrosis [2,14]. Faricimab’s simultaneous inhibition of Ang-2 and VEGF-A is thus designed to more comprehensively address these pathological processes, leading to enhanced vascular stability, reduced leakage, and potentially more sustained disease control compared to therapies targeting only VEGF-A [14,21,26]. Aflibercept, another anti-VEGF agent, also binds placental growth factor (PlGF) and VEGF-B, in addition to VEGF-A [10].

Preclinical studies have shown that combined Ang-2/VEGF-A inhibition resulted in a greater reduction in vascular leakage and inflammation compared to VEGF-A inhibition alone [2,14]. Supporting these preclinical findings, a clinical study by Todorok et al. demonstrated that switching nAMD patients from aflibercept to faricimab led to a significant decrease in aqueous humor Ang-2 levels one month after the first faricimab dose [30]. This reduction in Ang-2 correlated with central retinal thickness (CRT) improvement, providing clinical evidence of Ang-2 target engagement and its relevance to anatomical outcomes [30].

Pharmacokinetic data indicate an estimated vitreous half-life for faricimab of approximately 7.5 days in humans [2]. Systemic concentrations following intravitreal administration are low [21,22]. The systemic half-life of faricimab is about 7.5 days (or 89.3 h as reported in one source [22]), which is notably shorter than that of aflibercept (approximately 11.4 days) and bevacizumab (approximately 18.7 days) but similar to that of ranibizumab (approximately 8.5 days) [31]. Despite the Fc engineering aimed at reducing systemic impact, intravitreal faricimab administration has been shown to induce transient alterations in systemic plasma cytokine levels: plasma Ang-2 levels tend to increase, while plasma VEGF-A levels decrease, typically observed at 1 week post-injection, with levels trending back towards baseline by 4 weeks [22]. The rise in systemic Ang-2 may represent a counter-regulatory response or an escape mechanism [22]. The systemic VEGF suppression by faricimab appears less pronounced than that caused by aflibercept, which could be attributed to faricimab’s modified Fc region which limits FcRn-mediated recycling and thereby reduces systemic exposure [22]. This contrasts with aflibercept, which can significantly decrease systemic VEGF levels, while ranibizumab has no significant effect on systemic VEGF [9].

## 3. Efficacy of Faricimab in Neovascular AMD

The clinical efficacy of faricimab in nAMD has been robustly demonstrated in treatment-naïve patients through large Phase III clinical trials and further supported by real-world evidence and smaller studies in previously treated or treatment-resistant populations.

### 3.1. Treatment-Naïve Patients

The TENAYA [32,33,34,35] and LUCERNE [5,33,34,35] trials are the cornerstone Phase III studies for faricimab in treatment-naïve nAMD. These identical, global, randomized, double-masked trials compared faricimab 6.0 mg administered via a personalized treatment interval (PTI) regimen, allowing for dosing up to every 16 weeks (Q16W) based on disease activity assessments after four initial monthly loading doses, against aflibercept 2.0 mg administered every 8 weeks (Q8W) following three initial monthly loading doses [33,34,35]. The design and rationale for these trials aimed to assess efficacy, safety, and durability [33].

#### 3.1.1. Visual Acuity Outcomes

At the 1-year primary endpoint (mean change in BCVA from baseline averaged over weeks 40, 44, and 48), faricimab demonstrated non-inferiority to aflibercept Q8W [34]. In TENAYA, adjusted mean BCVA gains were +5.8 letters for faricimab and +5.1 letters for aflibercept [34]. In LUCERNE, both groups achieved +6.6 letters gain [34]. These visual acuity improvements were sustained through the second year of treatment; pooled 2-year data from TENAYA and LUCERNE showed adjusted mean BCVA changes from baseline of +3.7 letters for faricimab PTI and +3.3 letters for aflibercept Q8W [35]. Subgroup analyses generally aligned with global results. Patients from Asian countries enrolled in TENAYA and LUCERNE (1-year) showed mean BCVA gains of +7.1 letters with faricimab versus +7.2 letters with aflibercept [36]. The LUCERNE China subpopulation (1-year) reported comparable BCVA gains at weeks 40–48 (+9.7 letters for faricimab and +9.8 letters for aflibercept) [5]. The TENAYA Japan subgroup (1-year) also demonstrated similar efficacy, with BCVA gains of +7.1 letters for faricimab versus +7.7 letters for aflibercept [32]. Real-world data from the TRUCKEE study in Japan (treatment-naïve, n = 66) showed a gain of +8.6 ETDRS letters at 6 months [37]. Meta-analytic evidence further supports these results. A network meta-analysis by Butler et al. found faricimab T&E provided a +1.6 ETDRS letter gain (95% CI −0.6 to 3.8) compared to monthly ranibizumab at 12 months, with no anti-VEGF regimen demonstrating superiority over monthly ranibizumab in their comprehensive analysis [38]. An indirect treatment comparison using propensity score weighting was conducted by Galeone et al., who compared TENAYA/LUCERNE faricimab data with Italian real-world standard of care (SoC) data (aflibercept, bevacizumab, ranibizumab), and it suggested a greater mean BCVA gain of +5.4 letters for faricimab (*p* < 0.001) at 1 year [39].

#### 3.1.2. Anatomical Outcomes (CST, Retinal Fluid, PEDs)

Faricimab achieved robust reductions in central subfield thickness (CST) comparable to aflibercept Q8W at year one in TENAYA/LUCERNE [34]. Pooled CST reductions (averaged weeks 40, 44, 48) were −137.0 µm for faricimab and −130.1 µm for aflibercept [34]. These anatomical improvements were maintained at year two (faricimab −150.5 µm vs. aflibercept −142.3 µm, pooled) [33]. Subgroup analyses were consistent: the Asian country subgroup showed 1-year CST reductions of −129.9 µm (faricimab) and −128.1 µm (aflibercept) [36]; the LUCERNE China subpopulation reported 1-year CST reductions of −145.4 µm (faricimab) and −156.5 µm (aflibercept) [5]; the TENAYA Japan subgroup showed 1-year reductions of −140.6 µm (faricimab) versus −130.5 µm (aflibercept) [32]; and the TENAYA Japan subgroup showed reductions of −142.5 µm (faricimab) versus −144.5 µm (aflibercept) at 2 years [40]. Post hoc analyses of TENAYA/LUCERNE indicated that during the initial monthly head-to-head dosing phase (up to week 12), faricimab led to numerically greater CST reductions (−145.4 µm vs. −133.0 µm) and achieved absence of IRF and SRF faster (median time to fluid absence 4 weeks sooner) than aflibercept [14,41]. Faricimab also demonstrated greater reduction in both the presence (4% of patients with serous PEDs at baseline still had them with faricimab vs. 12% with aflibercept) and mean thickness (PED mean thickness was 27.9 µm thinner with faricimab) of serous PEDs at week 12 compared to aflibercept in these trials [14]. A study by Mukai et al. in treatment-naïve type 1 MNV patients showed that 3 monthly loading doses of faricimab resulted in significant PED maximum height reduction (from 240 ± 195 μm to 117 ± 112 μm at 3 months), which was comparable to the reduction seen with aflibercept [42]. Similarly, Fukuda et al. found comparable significant central macular thickness (CMT) reductions after three monthly loading doses of either faricimab or aflibercept in a propensity score-matched Japanese cohort of treatment-naïve nAMD patients [43]. Real-world data from Kunzmann et al. on 10 treatment-naïve eyes showed a mean CST reduction of −94.10 µm after one faricimab injection and −82.60 µm after three injections [44].

#### 3.1.3. Durability and Dosing Intervals

A defining feature of faricimab therapy is its potential for extended dosing intervals, reducing treatment burden [18,19,21]. In TENAYA/LUCERNE, at week 48, about 45% of faricimab-treated patients achieved a Q16W dosing interval, and approximately 80% (78–79%) were on ≥Q12W intervals [34]. By the end of the second year, around 60% of patients on faricimab were maintained on a Q16W schedule, and nearly 80% were on ≥Q12W dosing [35]. More than half of the patients who achieved Q16W at year one successfully sustained this interval at year two [35]. Durability was also robust in Asian patient subgroups: at week 48, 59.6% of the broader Asian country subgroup achieved Q16W dosing (91.2% ≥ Q12W) [36]; 67.3% of the LUCERNE China subpopulation achieved Q16W (87.3% ≥ Q12W) [5]; and in the TENAYA Japan subgroup, 66.1% were on Q16W (88.7% ≥ Q12W) [32].

### 3.2. Previously Treated/Treatment-Resistant Patients

To date, there is no universal consensus definition for “treatment-refractory” or “treatment-resistant” nAMD. In clinical practice and research, these terms are generally applied to cases with declined or suboptimal functional outcomes, persistent or recurrent intraretinal or subretinal fluid, inability to extend injection intervals, despite regular and adequate standard anti-VEGF therapy, most commonly with aflibercept or ranibizumab [37,44]. The utility of faricimab has also been investigated in these nAMD patients.

#### 3.2.1. Visual Acuity Outcomes

In this more challenging and heterogeneous group, VA outcomes vary. Many real-world studies have documented stable or slightly improved BCVA. Kaufmann et al. (273 switched eyes) reported stable logMAR VA at 12 months (0.47 baseline to 0.46) [45]. Kunzmann et al. (106 previously treated eyes) noted a +1.57 letter gain after 3 faricimab injections (*p* = 0.051) [44]. Cancian et al. (60 previously treated eyes) found a +2.2 letter gain at 12 months (*p* = 0.04) [46]. For treatment-resistant or refractory cases, some studies have shown more pronounced VA improvements. Rush (55 aflibercept-resistant eyes) documented a significant logMAR improvement from 0.57 to 0.41 at 12 months [47]. Tamiya et al. (47 aflibercept-refractory eyes) found logMAR BCVA improved significantly from 0.29 to 0.24 at 3 months [48]. Bleidißel et al. (48 poor responders to prior anti-VEGFs) reported a significant VA improvement from 0.54 to 0.40 logMAR over 4 faricimab injections [16]. Scampoli et al. (30 refractory eyes) reported significant BCVA improvement (0.77 to 0.62 LogMAR) over a 14.2  ±  1.9 months follow-up period [49]. However, not all studies demonstrate significant VA gains in this population. Cheng et al. (13 refractory eyes) reported stable VA after a mean of 3.7 faricimab injections despite significant anatomical improvement [7]. Kin et al. (15 eyes switched to faricimab) reported no significant BCVA improvement at 3 months compared to baseline [50]. Inoda et al. (80 previously treated eyes) found no significant BCVA change after one faricimab injection [51]. Jones et al. (52 previously treated eyes) documented no significant BMVA improvement after 6 faricimab injections [52]. Ng et al. reported no BCVA improvements in 63 previously treated eyes receiving a mean of 4.81 faricimab injections over 6.98 months [53].

#### 3.2.2. Anatomical Outcomes

Faricimab consistently demonstrates robust anatomical improvements in previously treated and treatment-resistant eyes. Significant reductions in CST and resolution of retinal fluid are frequently reported. Agostini et al.’s meta-analysis showed pooled CST reductions of −45.4 µm at 3 months and −41.6 µm at 12 months for those switched from aflibercept [29]. The TRUCKEE real-world study (521 eyes, predominantly previously treated) found that 49.9% of eyes showed retinal fluid reduction after a single faricimab injection, with a mean fluid volume reduction of −60.7 nL quantified by a deep learning algorithm; this fluid reduction was sustained with subsequent injections [25]. Hafner et al. (46 treatment-resistant eyes), also using an AI-driven OCT biomarker segmentation algorithm, documented significant median reductions in CRT (from 342.7 µm to 310.2 µm at 9 months), SRF, fibrovascular PED, and choroidal volume [15]. Cheng et al. (13 refractory eyes) reported a median CST reduction of 21.5 µm after 3 faricimab injections and a significant reduction in IRF/SRF height by 89 µm [7]. Scampoli et al. (30 refractory eyes) reported significant CST reduction (−57 μm on average) over a 14.2  ±  1.9 months follow-up [49]. Jones et al. (52 previous treated eyes) reported a moderate mean CFT decrease (from 274 ± 73 µm to 244 ± 59 µm) after six faricimab injections [52]. Numerous other real-world studies have confirmed these substantial anatomical benefits, including significant reductions in CST and various types of retinal fluid (IRF, SRF), leading to increased proportions of eyes achieving a dry macula [7,37,43,44,48,51,53,54,55,56,57,58,59,60,61,62,63]. For instance, Kaufmann et al. (273 switched eyes) showed CST decreased from 344 ± 128 µm to 296 ± 101 µm at 12 months, with dry macula rates increasing from 15.4% to 51.6% [45]. Ng et al. (63 previously treated eyes) demonstrated short-term anatomical response, as 25/63 eyes (39.1%) had complete dryness and 57/63 eyes (89.1%) had improvement in SRF/IRF after switching to the first dose of faricimab [53]. Prechoroidal clefts, considered a negative prognostic OCT biomarker, demonstrated variable responses to faricimab in a small case series, including persistence or recurrence in some instances [64]. Furthermore, a study by Todorok et al. showed that switching to faricimab significantly reduced aqueous Ang-2 levels, and this biochemical change correlated with CRT improvement [30].

#### 3.2.3. Durability and Dosing Intervals

A key goal of switching to faricimab in previously treated patients is to extend treatment intervals and reduce treatment burden. Many studies report success in this regard. Aldhanhani et al. (50 switched eyes) achieved a mean interval of 6.1 weeks after the third intravitreal faricimab injection [56]. Scampoli et al. (30 refractory eyes) reported that 90% of patients achieved treatment intervals of at least q8w, with 27% reaching q12w over a mean follow-up of 14.2  ±  1.9 months [49]. Jones et al. (52 pretreated eyes switched to faricimab) demonstrated an increase in the mean interval from 4.2 weeks to 5.8 weeks after six faricimab injections [52]. However, the ability to extend intervals is not universal. Some patients may not achieve significant extension, and a proportion (e.g., around 22–25% in some cohorts) might be switched back to their prior therapy due to perceived lack of efficacy or adverse events [15,54,65]. For example, Momenaei et al. found that switching patients with a suboptimal response to faricimab to aflibercept 8 mg did not typically result in interval extension or VA improvement, though CST did decrease [65]. Cases of suspected tachyphylaxis to faricimab, where its therapeutic effect diminishes over successive treatments, have also been reported, sometimes requiring a switch to a different anti-VEGF agent [66]. Factors associated with a poor response to faricimab in switch patients included lower baseline BCVA and higher prevalence of sub-RPE deposits like fibrovascular PEDs or subretinal hyperreflective material (SHRM) [61].

In resistant or previously treated nAMD eyes, real-world data show a consistent pattern: visual acuity is usually maintained, with modest improvement in selected cases, whereas anatomical responses such as CST reduction and fluid resolution are more robust [7,15,16,25,37,46,47,48,51,55,56,57,58,59,60,61,62,67]. These structural benefits often allow extension of treatment intervals, commonly from 4 weeks to 6–8 weeks or longer [49,52,56]. Overall, faricimab provides meaningful anatomical control and reduced injection burden in refractory cases, though functional recovery remains limited, making stabilization rather than major VA gain the realistic treatment goal.

## 4. Safety Profile of Faricimab in Neovascular AMD

Faricimab has generally demonstrated a favorable safety profile in nAMD patients, comparable to established anti-VEGF therapies like aflibercept 2 mg, across its extensive clinical development program and in emerging real-world use.

### 4.1. Intraocular Inflammation (IOI) and Vasculitis

#### 4.1.1. Phase III Trials (TENAYA/LUCERNE, nAMD)

In the 1-year primary analyses, the pooled incidence of IOI was 2% (13/664 eyes) for faricimab and 1.2% (8/662 eyes) for aflibercept [34]. Over two years, the cumulative IOI incidence for faricimab was 3.3% in TENAYA and 2.7% in LUCERNE, compared to 1.5% (TENAYA) and 3.1% (LUCERNE) for aflibercept [35]. Most IOI events were reported as mild to moderate and typically resolved with topical corticosteroids [34,35]. No cases of retinal vasculitis or occlusive retinal vasculitis were reported in the faricimab arms through two years of TENAYA/LUCERNE or in the subsequent AVONELLE-X extension study up to Week 160 [34,35].

#### 4.1.2. Real-World Evidence

Reported IOI rates in previously treated/switched patient cohorts generally range from 0% to 4.0% [16,37,40,43,44,48,50,51,56,57,58,61,62,63]. Bourdin et al. reported an overall IOI incidence of 0.87% per faricimab injection in their center, with a specific pattern of vitritis (dense, grayish vitreous bands) occurring in 0.63% of injections; they noted this was higher than other anti-VEGFs used in their center during the same period [68]. Janmohamed et al. described a series of 5 patients who developed severe IOI with features suggestive of HSV keratouveitis following faricimab therapy, positing a possible link between faricimab’s dual Ang-2/VEGF-A inhibition and altered ocular immune surveillance facilitating viral reactivation [69]. Thangamathesvaran et al. detailed 3 cases of acute, severe, initially culture-negative posterior segment IOI (1.6% of injections in their cohort) occurring within a short period at their institution [70]. Most reported real-world IOI cases are mild anterior uveitis and resolve with topical steroids [39,48,56,58,61,62].

#### 4.1.3. FDA/Manufacturer Warning (Nov 2023)

Following post-marketing surveillance, a drug warning was issued acknowledging rare reports of retinal vasculitis and/or retinal vascular occlusion associated with faricimab [69,71,72]. Genentech released a drug warning stating that the estimated reporting rate was 0.06 per 10,000 injections for retinal vasculitis with occlusion and 0.17 per 10,000 injections for retinal vasculitis with or without occlusion [73]. These rates are notably lower than those reported for brolucizumab but higher than historical rates for aflibercept 2 mg [74,75].

#### 4.1.4. Subclinical Inflammatory Changes

Matsubara et al. found a transient, slight increase in aqueous flare values (AFV) two weeks after faricimab injection (median change +4.60 pc/ms), which was greater than that observed with aflibercept or brolucizumab [28]. This was not associated with clinical IOI and was hypothesized to be potentially due to the diffusion of the larger faricimab molecule into the anterior chamber rather than representing true inflammation [28].

### 4.2. Retinal Pigment Epithelium (RPE) Tears

The incidence of RPE tears in TENAYA/LUCERNE at 1 year was low and comparable between groups (faricimab 0.3%, aflibercept 0%) [34]. Cumulative 2-year rates also remained low and comparable (faricimab 0.6%, aflibercept 0%) [35]. RPE tears were often observed in eyes with large baseline PEDs [14]. The LUCERNE China subpopulation reported one case of a mild RPE tear (1.7%) with faricimab [5]. Real-world studies and case reports note RPE tear rates between 0.7% and 3.6% in various cohorts, including switch patients [26,56,57,63]. Mukai et al. observed RPE tears in 2 out of 18 (11%) treatment-naïve eyes with type 1 MNV after faricimab loading doses; these eyes had particularly high baseline PEDs [42]. The authors noted this was not statistically different from their aflibercept cohort (0 RPE tears) [42]. A case report also highlighted RPE tear development after switching from aflibercept to faricimab in an eye with persistent disease activity and a large PED [76].

### 4.3. Intraocular Pressure (IOP)

Transient IOP elevations immediately post-injection are a known class effect of all intravitreal therapies, primarily due to the injected volume [77]. A prospective comparative study by Paris et al. found that faricimab (0.05 mL administered from a vial) resulted in a statistically significantly smaller mean IOP spike at 30 s (+32.19 mmHg) compared to aflibercept 2 mg (0.05 mL administered via a pre-filled syringe, resulting in a spike of +41.47 mmHg) and aflibercept 8 mg (0.07 mL from vial, +43.46 mmHg). However, these inter-group differences in IOP elevation were no longer significant by 5–15 min post-injection, as mean IOPs returned to normal limits for all treatment groups [77]. Regarding pre-injection IOP measurements at subsequent visits during the loading phase, Honjo et al. found no significant change from baseline IOP in the faricimab group at 3 months (baseline 13.8 ± 2.1 mmHg to 13.5 ± 1.5 mmHg), while the aflibercept group showed a small significant decrease in their study of treatment-naïve Japanese nAMD patients [23].

### 4.4. Endophthalmitis

The rate of endophthalmitis in TENAYA/LUCERNE at 1 year was rare and identical between faricimab (0%) and aflibercept (0.2%) [34]. Stevanovic et al.’s NMA of Phase III trials reported an endophthalmitis incidence of 0.8% for faricimab [27]. We summarize the reported proportion of patients experiencing ocular AEs with intravitreal faricimab for nAMD in RCT and real world studies (Table 1).

### 4.5. Systemic Safety

#### 4.5.1. Systemic Cytokine Levels

Intravitreal faricimab injection leads to a transient increase in systemic plasma Ang-2 levels and a transient decrease in systemic plasma VEGF-A levels 1 week post-injection, with levels generally returning towards baseline by 4 weeks [22]. This systemic VEGF suppression by faricimab is generally less pronounced than with aflibercept [22].

#### 4.5.2. Renal Adverse Events

A comprehensive meta-analysis by Huang et al., including faricimab, found no significant increase in acute kidney injury (AKI) risk for FDA-approved anti-VEGFs compared to sham or other anti-VEGFs. Faricimab’s AKI incidence was 0.8%; patient-reported renal symptoms were low (0.1%) [78].

#### 4.5.3. Arterial Thromboembolic Events (APTC-Defined)

Pooled TENAYA/LUCERNE data at 1 year showed comparable low rates: 1% for faricimab, 1% for aflibercept [34]. Rates slightly increased but remained similar between these two agents at 2 years (3% for faricimab, 3% for aflibercept) [35].

## 5. Discussion

Faricimab’s introduction into the therapeutic arsenal for nAMD signifies a notable advancement, primarily due to its unique dual mechanism of action targeting both VEGF-A and Ang-2 [14,21,24]. This dual inhibition strategy is predicated on the understanding that nAMD pathogenesis involves more than VEGF-A alone, with Ang-2 recognized as a key contributor to vascular destabilization, inflammation, and fibrosis [2,14]. The large Phase III TENAYA and LUCERNE trials provide robust evidence of faricimab’s non-inferiority to aflibercept 2 mg Q8W in terms of visual acuity gains in treatment-naïve patients over two years and beyond [34,35]. This efficacy is supported by significant anatomical improvements, including CST reduction and resolution of retinal fluids, with some analyses suggesting faster initial fluid control and greater PED reduction with faricimab [14,79,80]. The most significant clinical advantage highlighted by many trials is faricimab’s extended durability; the majority of patients in these trials successfully maintained extended dosing intervals of Q12W or Q16W, significantly reducing the annual injection frequency and associated treatment burden [34,35]. These positive outcomes have been consistently observed across diverse global populations, including specific analyses of Asian, Japanese, and Chinese patient subgroups, where durability might even be enhanced [5,32,35,40].

In the context of previously treated or treatment-resistant nAMD, a growing body of real-world evidence indicates that switching to faricimab often results in significant anatomical improvements, particularly in CST reduction and fluid resolution [7,15,16,25,37,46,47,48,51,54,55,56,57,58,59,60,61,62,63,67]. Visual acuity in these challenging cases often stabilizes or shows modest gains [46,47,48]. Importantly, treatment intervals can frequently be extended, offering relief from intensive treatment schedules [56,57]. The observed reduction in aqueous Ang-2 levels post-switch, which correlates with anatomical improvement, provides a biological rationale for these benefits by confirming active engagement of the Ang-2 pathway [31].

The short-term efficacy of faricimab—whether evaluated by visual outcomes or anatomical response after loading doses—has been consistently demonstrated in pivotal trials [32,33,34,35,36]. In real-world practice, where loading regimens typically involve three or four monthly injections, comparable efficacy has also been reported [37,44,81,82,83]. Mukai et al. and Matsumoto et al. observed significant BCVA improvement and CRT/CFT reduction in treatment-naïve eyes, with 82% and 79.5% of eyes achieving a dry macula after the loading phase [81,83]. The TRUCKEE study, which mainly involved previously treated eyes, reported a mean BCVA gain of +3.4 letters and a mean CST reduction of −43.4 µm following three consecutive injections [37]. Similarly, Yufeng et al. (in 35 previously treated eyes) demonstrated three consecutive monthly injections significantly improved BCVA (logMAR 0.84 ± 0.49 to 0.76 ± 0.49) and reduced CMT (447.65 ± 161.46 µm to 327.33 ± 147.78 µm) [82]. By contrast, Doi et al. found no significant differences in functional-anatomical outcomes between treatment-naïve eyes receiving either three or four monthly loading doses [84].

While these early responses are clinically meaningful—particularly as predictors of macular drying and treatment interval extension—they raise the important question of whether such benefits can be sustained. Indeed, several studies have reported short-term outcomes (3–6 months) showing functional and anatomical gains [7,16,25,48,50,51], yet the durability of these effects requires confirmation in longer-term cohorts. Longer-term series extending to 12 months or more indicate that structural benefits such as CST reduction and fluid resolution can be maintained, although visual improvement varies [45,46,47,49]. These observations suggest that short-term improvements may not always translate into long-term benefits, especially in refractory cases. Accordingly, both short-term induction effects and long-term maintenance data should be considered carefully when assessing the clinical utility of faricimab.

OCT biomarkers play a central role in tailoring individualized treatment intervals, monitoring disease activity, and evaluating the efficacy of anti-VEGF treatment for nAMD [3,12,33,34]. Nanji et al. assessed the prognostic value of 80 baseline OCT biomarkers in anti-VEGF–treated nAMD; the presence of IRF and SHRM was associated with poorer visual outcomes at 12 months, whereas intact EZ and ELM predicted better outcomes. However, the certainty across all 80 biomarkers remained low [85]. In faricimab-treated nAMD, both real-world data and pivotal trials consistently reported that reductions in CST and resolution of IRF/SRF were accompanied by improvements in VA [15,33,34,35,37,43,44,46]. Nonetheless, the strength of this trend appears context dependent: it is more robust in treatment-naïve eyes, whereas in previously treated/refractory eyes the relationship is more variable [33,34,35,36,44,45,46,47]. In treatment-naïve eyes receiving faricimab, Scampoli et al. reported that an early reduction in CST and early achievement of a dry macula were indicative of effective disease control and the potential for longer treatment intervals, respectively [86]. Hafner et al. demonstrated that the resolution of IRF and SHRM correlated most strongly with improvements in BCVA [87]. In previously treated eyes, higher baseline CRT and fibrovascular PED may indicate greater visual improvement and the potential for treatment interval extension [15]. As OCT biomarker play a big role in nAMD treatment, AI offers a way to enhance their clinical use.

AI and deep learning are increasingly recognized as valuable tools for optimizing faricimab therapy in nAMD, primarily through the analysis of OCT imaging [15,25,79,80,87,88]. Building on this, Hafner, Asani and colleagues introduced a deep-learning–based segmentation algorithm that enables automated, objective, and standardized quantification of OCT biomarkers—including identification, localization, and volumetric measurement of IRF, SRF, SHRM, hyperreflective foci (HRF), PED, and thickness metrics [15,87,88]. Kikuchi et al. utilized data from a phase II trial to train machine learning models, aiming to predict visual and anatomical outcomes in treatment naïve patients receiving faricimab; the models showed moderate accuracy, with baseline BCVA and CST being the strongest predictors [89]. These findings suggest that AI can help identify patients most likely to benefit from faricimab, support individualized treatment based on disease activity, and potentially reduce overtreatment while improving long-term outcomes [15,87,88,89]. To date, these AI approaches are based on small cohorts and require validation in larger real-world studies before routine clinical adoption.

The safety profile of faricimab is a critical aspect of its clinical utility. Overall, faricimab has been shown to be well-tolerated, with a safety profile generally comparable to aflibercept 2 mg [34,35]. IOI rates in clinical trials were low, although some reports showed slightly higher numerical incidences for faricimab in certain analyses [34,35]. Real-world reports have highlighted varying IOI incidences, with some centers observing higher rates or distinct patterns like vitritis [68]. The potential for HSV reactivation in a few IOI cases is an area requiring further research [69]. Importantly, retinal vasculitis and related occlusive events were not reported in the faricimab arms during the nAMD Phase III trials [34,35]. The subsequent FDA/manufacturer warning regarding very rare post-marketing reports of these events (0.06–0.17 per 10,000 injections) highlights the importance of pharmacovigilance, though the reported rates are lower than for brolucizumab [73,74,75]. Other ocular adverse events like RPE tears and endophthalmitis are rare and occur at rates comparable to other anti-VEGFs [27,34,35]. Systemic safety appears favorable, with no increased risk of AKI and potentially fewer overall SAEs than aflibercept, possibly due to its engineered Fc region minimizing systemic exposure [22,27,78].

The ability of faricimab to achieve durable disease control with fewer injections has significant implications for reducing treatment burden [18,19,20]. This is particularly important given the rising prevalence of nAMD and the strain on healthcare systems [20]. Cost-effectiveness analyses support the value of faricimab, especially when societal costs related to fewer visits are factored in [20,90]. The TANGO treatment regimen, designed for newer generation anti-VEGFs like faricimab, aims to optimize treatment schedules and further reduce clinic visits [18].

While current evidence strongly supports faricimab’s role in nAMD, continued long-term real-world data collection is essential to fully delineate its sustained effectiveness and safety profile across diverse patient populations and clinical scenarios. Comparisons with other newer long-acting agents, such as aflibercept 8 mg, are also emerging from network meta-analyses [91]. Such analyses suggest comparable efficacy between faricimab and aflibercept 8 mg, with aflibercept 8 mg potentially requiring slightly fewer injections in the first year in some nAMD populations [91]. Case reports where patients were switched between these newer agents highlight the individualized nature of responses and the complexities in managing refractory disease [7,65,92,93].

Based on current evidence, faricimab offers flexibility across the nAMD treatment spectrum. In treatment-naïve patients, consistent efficacy and extended durability support its consideration as a first-line option when long-term burden reduction is a priority. In previously treated eyes, particularly those with high injection demand or incomplete control, switching to faricimab may yield anatomical improvement and interval extension, though visual gains are often modest. The safety profile remains broadly comparable to other anti-VEGFs, and continued vigilance is appropriate. By lowering visit frequency and potentially improving adherence, faricimab provides practical value, while ongoing real-world data will refine its role in clinical care.

## 6. Conclusions

Intravitreal faricimab, by dually inhibiting VEGF-A and Ang-2, represents a significant therapeutic advancement for neovascular AMD. In treatment-naïve patients, it delivers robust visual and anatomical outcomes non-inferior to aflibercept 2 mg, with the significant advantage of extended dosing intervals up to Q16W or longer for the majority of patients. For many individuals previously treated with or refractory to other anti-VEGF therapies, faricimab frequently leads to improved anatomical control and offers the possibility of interval extension, often with stable or modestly improved vision. The overall safety profile of faricimab is generally favorable and similar to that of other anti-VEGF therapies. However, ongoing pharmacovigilance for rare adverse events like severe IOI and retinal vasculitis is essential. By reducing treatment frequency, faricimab has the potential to lessen the substantial burden of nAMD management for patients, caregivers, and healthcare systems, making it a valuable addition to the therapeutic armamentarium.

## Figures and Tables

**Table 1:** Reported proportion of patients experiencing ocular AEs with intravitreal faricimab in nAMD.

	RCTs (TENAYA/LUCERNE)		Real-World
	1-Year Analysis	2-Year Analysis	
IOI	2%	2.7–3.3%	0–4%
RPE tears	1%	0.6%	0.7–3.6%
IOP increase	<1%	0–0.3%	Not Applicable *
Endophthalmitis	0%	0.3–0.6%	Not Applicable *

* Data on IOP increase and endophthalmitis are lacking in real-world studies.

## Data Availability

Not applicable.
